# Practical Items, or a Chapter on Small Matters

**Published:** 1854-10

**Authors:** 

**Affiliations:** Chicago


ART. II.—*Practical Items, or a Chapter on small matters.* By one of the Editors.

The Physician in his daily round of practice frequently meets with ailments, which though not dangerous to the life of the patient, are nevertheless exceedingly annoying to him, and sometimes perplexing to the Physician. Hence a few cases and observations concerning small matters may not be uninteresting to our readers.

CASE 1ST. *Onychia.* Called to see Mrs. A. who had been sorely afflicted for many weeks with pain and soreness in one large toe. Found the whole toe swollen and tender, but the chief trouble was limited to one side, extending to one half of the circumference of the nail. The corner of the nail next to the first little toe was deeply imbedded in the flesh, giving it the appearance of being much incurved. All along that margin the flesh much overlapped the nail, was swollen, covered with red and spongy granulations, and secreted a thin and redish purulent matter. This was so tender that walking was accompanied by much pain. She had consulted a Physician who, as is usual in such cases, told her the only remedy consisted in extracting, or cutting out a considerable portion of the nail. Dreading the pain of such an operation she begged of me some other remedy. I directed her to place the foot up in a horizontal position and cover the toe with a soft linseed meal poultice for twenty-four hours, to lessen the inflammation. I then removed the poultice, wiped it dry and covered the side of the toe with collodion in such a way that its contraction should hold the overlapping flesh and granulations away from the nail, and renewed its application often enough to keep the coating perfect. I caused her also to drop, twice a day, a few drops of a solution of Bi Chloride of mercury in lime-water, into the fissure between the nail and overlapping granulations. The strength of the solution was as follows, viz:

℞ Bi Chloride of Mercury - 2 grs.
Aqua Calcis - - - ℥ij—mix.

This treatment was continued five or six days, during which time the swelling diminished, and the granulations overlapping the nail receded very much. It being troublesome to keep the coating of collodion perfect, this was now omitted. The same wash was continued once a day, and during the rest of the time a salve was applied of Chloride of Zinc 3 grs
Sulph. Morphine 6 grs
Simple Cerate ℥j, thoroughly rubbed together and spread on soft linen. In one week more the toe was entirely well; the flesh having receded from over the nail and cicatrized so completely as to have neither discharge nor soreness, The nail was in good shape, and by avoiding all undue pressure of the shoe for a few weeks, no return of the disease took place, and now at the end of two years the toe is perfectly well.

During the last five years I have treated quite a number of cases of this troublesome malady, and in every instance have succeeded in effecting a cure without removing any part of the nail. I have treated them on the same principles that I treat ulcers. In recent cases where the part is very irritable and painful, I apply for two or three days emolient poultices. Then wash the part overlapping the nail carefully once or twice a day with a weak solution of Sulphate of Copper, or Bi Chloride of Mercury in lime water, or Chloride of Zinc in water. The latter, as a wash, should be of the strength of 1 gr. to the oz of water.

After the poultices are removed I keep the parts protected by soft cloth spread with the salve mentioned above. The cure is generally completed in from one to three weeks. The patient should always be cautious about putting on tight boots or shoes for sometime after the toe appears to be well. The common idea that the nail is incurved and grown down into the flesh, in such cases is erroneous, and the operation of cutting it out unnecessary. The disease is generally a simple inflammation, commencing at the edge of the root of the nail. When once begun the swelling immediately causes the flesh to commence overlapping the edge of the nail and the pressure of the adjoining toe and shoe keeps it constantly aggravated. Thence it becomes painful, the granulations large and spongy, and the pus discharged generally thin and intermixed with blood. The same disease sometimes occurs on the fingers as in the following case:

Case 2nd. Mrs. B., a hard working Irish woman, came under my care with disease of one thumb and three fingers. The husband was consumptive and she was in the habit of supporting the family by washing and house-cleaning.

The disease on the thumb and fingers commenced like an ordinary inflammation at the corner of the nail. It extended rapidly across the root of the nail, causing, in a few days, the formation of a few drops of pus, over which the skin became elevated and white, like a mature pustule. The parts became much swollen, red, and very painful. A prick through the elevated cuticle gave exit to a few drops of thick whitish pus, but this instead of being followed by an abatement of the inflammation and pain, was marked by a continuance of both, and the formation of spongy granulations overlapping the root and side of the nail, much like the diseased toe already described.

When she came under my care, different fingers presented the disease in different stages of progress. She also had several superficial and very irritable ulcers on the inner margin of the Labia. These had not the appearance of syphilitic sores or chancres, but looked more like simple excoriations. Neither could I learn that she had ever had syphilis in any form.

The tongue of the patient was coated and her general health somewhat impaired, in consequence of which I directed her a few grains of Blue Mass and Soda each night for two or three evenings, and then gave five grains of Iodide of potassa in a teaspoonful of tincture of Cinchonæ three times a day. Locally to the fingers, I made the same applications as already detailed in reference to case 1st. and to the Labia I directed a wash of chloride of zinc and morphine, and the application of a weak mercurial ointment at bed-time. The fingers gradually improved and at the end of two weeks, were quite well; but the labia remained swollen, painful and covered with irritable ulcers. These were apparently kept up by the friction of walking, and yet such were the circumstances of the patient that entire rest was almost impossible. Hence to shield the parts from friction, they were made dry and coated over completely with collodion. The application of the collodion to the ulcerated surface caused acute smarting for a few minutes, after which she experienced much relief so long as the coating remained perfect. But the moisture of the parts would cause it to become loose, and make it necessary to renew the application daily. Under this treatment, the swelling and pain disappeared from the labia in five or six days, and the ulcerations soon healed. She had at no time any luccorrhœal discharge.

Case 3d *Phagdenic ulcer* May 1851, was called to see a little girl, aged five years. She looked slender and delicate, with thin, fine hair, light eyes, and fair skin The mother represented her general health as being very good, but the end of one thumb had been diseased nearly one year. It commenced, as near as I could learn, like a pustular inflammation at the root of the nail.

But it soon became an open sore and continued to spread until the time of my first visit. Then the whole end of the thumb was enlarged with a ragged concave ulcer occupying the place of the thumb nail; the latter being entirely destroyed. The edges of the ulcer were irregular, but elevated and rounded; the surface of the ulcer without granulations and emiting a thin, sanious, or bloody matter, somewhat offensive to the smell. The end of the thumb so far as it was enlarged felt inelastic and doughy, the capillary circulation seemed feeble, and it was constantly perspiring or oozing from the pores of the skin a colorless watery fluid. This state of the capillaries and skin was limited exclusively to the pulp and enlarged end of the thumb.

As various local applications had been tried without any benefit, and the disease had existed a long time, I thought it best to put the patient on some constitutional treatment.

I accordingly directed for her five drops of Fowler's Solution of arsenic in a wine-glass full of sweetened water every morning. I also caused the ulcer to be washed clean every morning wet over with a solution of chloride of zinc of the strength of 1 gr to the oz. of water, and covered with a salve spread on soft cloth, composed of chloride of zinc 4 grs. sulph. morph. 6 grs. and simple cerate ℥j. The ulcer soon began to improve in its appearance. The arsenical solution was taken only little more than one week, but the local applications were steadily continued four or five weeks, during which time the ulcer healed, and the thumb has since become perfectly well and natural, except the nail, which has been only imperfectly reproduced.

CASE 4TH. A Swede girl, aged 12 years, was brought to me from Wisconsin. She presented a forbidding appearance. She was extremely emaciated, the skin of a dingy color and dry; the soft part of the nose was entirely gone and in its place an open irregular ulcer. The lips, too, were nearly gone, and the same kind of ulcer occupying the whole circumference of the mouth.—The central part of the upper lip was entirely removed leaving the teeth and gums bare. On the left leg near the knee joint, over the upper end of the fibula, was an ulcer about the size of a half dollar coin, and another still larger just above the outer maleolu of the ankle. The tendons of the limb were somewhat contracted, rendering the patient unable to walk. All the ulcers were superficial with irregular but not elevated edges. They seemed to spread by destroying the tissues without much formation of pus, and without much pain. Her bowels were regular and appetite good, though suffering great general debility and emaciation. There was no appearance of present or previous disease of the throat.

Speaking only the Swedish language, I could learn from the father very little in reference to the history of the family.

The mother had been dead some years, but the father bore plain evidence in his fauces of having had secondary syphilis which was even now imperfectly cured. Believing the girls sickness to depend on a *hereditary* syphilitic influence, I directed for her the following, vis.:

℞ Bichloride Hydrarg. 2 grs.

Tincture Cinchona. ℥jv– mix.

Give one tea-spoonful (fl. ʒi) three times a day in a little sweetened water. Also keep the ulcerated surfaces clean and dressed every day with the following ointment spread on soft linen, viz. :

℞ Chloride of Zinc, 4 grs.
Sulph. Morph. 6 grs.
Simple Cerate, ℥j rub thoroughly together.

In four or five days the ulcers began to show marks of improvement, and from that time they cicatrized rapidly.

In about two weeks the gums began to show signs of being affected by the mercurial, and it was discontinued. In its place I gave four grains of Iodide of potassa with a tea-spoonful of Tinct. Cinchonæ three times a day for two weeks. She then took again the first prescription in smaller doses, and in two or three weeks more her ulcers, both on the face and leg, had entirely healed. She had also gained much in flesh and strength, and could walk about very well. She was now put upon the use of the Iodide of Potassa with tincture of bark in moderate doses, and a nutritious but not stimulating diet, and allowed to return home The local application first directed was continued during the whole period of the treatment. The father promised if she did not continue well, to inform me; but I have heard nothing from him, nearly six months having now elapsed since he left. I have had several cases similar to the above brought to me from the country, which have uniformly yielded to essentially the same course of treatment. It is always necessary to watch the action of the Bichloride of mercury on the bowels, as it sometimes produces hypercatharsis with very severe griping pains. This can be obviated, when necessary, by giving an anodyne of Pulv. Doveri, at bedtime.

While on the subject of ulcers I will relate one more case which came under my observation very recently:

Case 5. Mrs. G., a slender Irish girl had been married within the last 18 months, but had not yet been pregnant. Her husband was intemperate and abusive, and she lived in misery and privation. About three months since she was taken sick, but I could not learn enough of the history of her case to judge with certainty in regard to its true nature. She had considerable general fever in the beginning, accompanied by pain in her back and a very offensive leucorrhœal discharge. The discharge from the vagina ceased after a few days, but she continued feverish and much unwell. I could not learn much of the particular symptoms, but she had no sore throat,no bubos, and no ulcers or chancres about the labia. At the time of my visit there was no disease whatever about the genital organs, but she was extremely emaciated, scarcely able to turn herself in bed, without appetite, and suffering much pain from two or three bedsores over the sacrum, and a large phagadenic or eating ulcer in one groin. The latter first manifested itself by a purple spot which marked the seat of a slough or eschar. This separated and left a deep, irregular and spreading ulcer, accompanied by very severe pain At the time of my visit the ulcer was about the size of a silver dollar in circumference, from a quarter to half an inch in depth, the edges excavated and irregular, and the bottom destitute of granulations. The bowels were nearly regular, the tongue clean and the appetite moderate. She was removed to the Mercy Hospital, and I put her upon the use of the following, viz.:

℞ Proto-Iodide of Mercury, 10 grs.

Pulv. Opii - - 10 grs. mix, divide into twenty pills. Gave one each morning, noon, and night. Caused the ulcers both on the back and in the groin to be washed twice a day with the following solution:

℞ Chloride of Zinc. 4 grs.

Sulph. Morph. 12 grs.

Water - ℥jv mix—and dressed with Simple Cerate. She was allowed a plain nutritious diet, consisting of milk-porridge, (sweet milk boiled with flour), tenderly cooked lean meat, and stale bread She continued this treatment about ten days, with a rapid improvement in all her symptoms. The ulcers ceased to be painful and began to fill up with healthy granulations. The Proto Iodide of mercury was then discontinued, and the tincture of Ferri Murias, in doses of 20 drops, three times a day, given in its place. The same local applications were continued as at first. Under this course the patient continued to improve, and in three weeks from the time of her admission the ulcers had entirely cicatrized, and the patient had recovered a good degree of flesh and strength.

Chicago, October 1854.

